# Whole Genome Amplification and *De novo* Assembly of Single Bacterial Cells

**DOI:** 10.1371/journal.pone.0006864

**Published:** 2009-09-02

**Authors:** Sébastien Rodrigue, Rex R. Malmstrom, Aaron M. Berlin, Bruce W. Birren, Matthew R. Henn, Sallie W. Chisholm

**Affiliations:** 1 Department of Civil and Environmental Engineering, Massachusetts Institute of Technology, Cambridge, Massachusetts, United States of America; 2 The Broad Institute of MIT and Harvard, Cambridge, Massachusetts, United States of America; University of Hyderabad, India

## Abstract

**Background:**

Single-cell genome sequencing has the potential to allow the in-depth exploration of the vast genetic diversity found in uncultured microbes. We used the marine cyanobacterium *Prochlorococcus* as a model system for addressing important challenges facing high-throughput whole genome amplification (WGA) and complete genome sequencing of individual cells.

**Methodology/Principal Findings:**

We describe a pipeline that enables single-cell WGA on hundreds of cells at a time while virtually eliminating non-target DNA from the reactions. We further developed a post-amplification normalization procedure that mitigates extreme variations in sequencing coverage associated with multiple displacement amplification (MDA), and demonstrated that the procedure increased sequencing efficiency and facilitated genome assembly. We report genome recovery as high as 99.6% with reference-guided assembly, and 95% with *de novo* assembly starting from a single cell. We also analyzed the impact of chimera formation during MDA on *de novo* assembly, and discuss strategies to minimize the presence of incorrectly joined regions in contigs.

**Conclusions/Significance:**

The methods describe in this paper will be useful for sequencing genomes of individual cells from a variety of samples.

## Introduction

Genome sequencing is one of the most powerful tools for accessing the genetic and metabolic diversity found in microorganisms. However, the vast majority of microbes elude cultivation [1], making complete genome sequencing impossible using traditional approaches. Genome sequencing of individual cells, enabled by whole genome amplification (WGA), presents a new, culture-independent approach for exploring the genetic diversity and evolutionary history of microbes [2]. Sequencing complete genomes from single cells will, for example, allow the exploration of the uncultured microbial majority [3]–[5] and facilitate analysis of cell-to-cell variability in microbial populations. If applied at large enough scales, the strategy could also be highly complementary to metagenomics studies, filling important gaps in our understanding of how genetic diversity found within microbial communities is discretely organized into co-existing cells. With the recent advances in sequencing technologies, sequencing individual microbial genomes at significant scales will soon be a reality [6]. The main challenges at this point are to develop robust, high-throughput pipelines to generate high-quality sequencing libraries from single cells, and to establish a thorough understanding of the potential pitfalls of the approach.

A typical bacterial cell contains only a few femtograms of DNA, making whole-genome amplification (WGA) necessary for sequencing whole genomes from single cells using current technologies [7], [8]. Multiple displacement amplification (MDA) with bacteriophage Phi29 DNA polymerase is emerging as the preferred method for WGA, as it can amplify minute quantities of DNA by several orders of magnitude with very high fidelity, producing fragments that are tens of kilobases long [2]. However, several characteristics of MDA constitute challenges for single-cell genome sequencing. First, the reaction relies on random primers to initiate polymerization [2], [9], resulting in the amplification of both target and contaminating DNA[8]. This can pose a significant problem when investigating novel organisms, as non-target sequences could be incorrectly ascribed to the target organism. Second, MDA produces large biases in sequencing libraries, leaving some regions of the genome orders of magnitude more abundant than others [2], [10], [11]. This phenomenon appears more pronounced as smaller amounts of DNA are used as a template for amplification, and significantly increases the sequencing effort required to achieve sufficient coverage across the whole genome. Finally, MDA produces genomic rearrangements, or chimeras, that can complicate genome assembly by linking non-contiguous chromosomal regions [2], [11].

The isolation of individual cells for WGA also presents difficulties. Ideally, such a procedure should (1) allow hundreds to thousands of cells to be processed rapidly; (2) ensure that no more than one cell is included in each amplification reaction yet maximize the number of cell-containing reactions; and (3) minimize contaminating DNA. Several strategies for single-cell isolation have been reported, including serial dilution [11], microfluidics [10], and micromanipulation [3]; none of these, however, currently satisfies all three conditions. By comparison, fluorescence-activated cell sorting (FACS) has several key features that make it particularly well-suited for high-throughput, single-cell WGA [12]: it allows individual cells to be deposited accurately and rapidly into standard 96- or 384-well plates [13], [14], which are compatible with automated liquid handlers for setting up WGA reactions; information about cells such as their size, pigmentation, cell membrane characteristics, and genetic composition can be measured and used to select for cells within specific populations [14]; and cells are sorted in tiny droplets containing only a few picoliters of initial sample solution, reducing the co-transport of contaminating DNA [12], [15].

In recent years, partial genome sequences of uncultured bacteria found in soil [3], coastal ocean waters [5], and the human mouth [4] have been generated using single-cell WGA, offering important insights into the metabolic potential of these microbes. However, it is difficult to address with complete confidence questions regarding contamination, chimeras, and genome recovery when examining uncultured organisms that lack a reference genome. Rigorous examination of single-cell genome amplification and assembly with a previously sequenced microbe is therefore essential to evaluate both the challenges and possibilities of single-cell genomics, and to help lay a methodological foundation for the investigation of uncultured microbes.

We used the marine cyanobacterium *Prochlorococcus* as a model to address the central challenges facing the development of large-scale single-cell genomics. *Prochlorococcus* represents an ideal system for further development of single-cell genomic technologies. Several isolates have already been sequenced and have relatively small genomes with few repetitive regions [16], [17], facilitating sequencing and assembly, as well as allowing for a more rigorous evaluation of methodologies'performances. Second, *Prochlorococcus* cells can be easily identified and sorted by FACS, due to their unique autofluorescence and light scatter signals [18]. Finally, *Prochlorococcus* is the most abundant photosynthetic organism in the ocean [19], and its gene sequences often dominate oceanic metagenomic libraries [20]–[22] – making it a prime target for future large-scale single-cell genomics studies.

## Results

### High throughput single-cell WGA

We developed a pipeline for single-cell WGA that utilized FACS to rapidly deposit individual cells in standard 96- or 384-well plates, and semi-automated liquid handling to set up MDA reactions. This approach enabled MDA on hundreds of individual *Prochlorococcus* cells at a time, of which ∼40% of the reactions were successful as determined by PCR screening with universal primers targeting the 16S-23S internal transcribed spacer.

Minimizing free DNA, which can be introduced to MDA reactions through co-transport with cells, can be particularly challenging with environmental samples such as seawater, in which the amount of extracellular DNA (∼5 ng/mL) can equal or exceed the amount of DNA contained within cells [23]. In principle, multiple rounds of FACS should dilute free DNA to insignificant levels. To confirm this hypothesis, a culture of *Prochlorococcus* MED4 was intentionally contaminated with known DNA sequences (5 ng/mL) prior to sorting and MDA. Two cycles of FACS were then performed to dilute the introduced DNA by an estimated 9 orders of magnitude before performing MDA. Over 5,000,000 Illumina reads were generated from two replicate single-cell amplified genomes (SAGs), but neither SAG contained sequencing reads matching the contaminating DNA.

Strict handling procedures were also used to keep DNA contamination levels low while maintaining high throughput. Total background contamination levels ranged from 10^−2^–10^−4^ fg DNA per reaction (Figure S1), low enough to make amplification of contaminating DNA negligible. Greater than 99% of all 454-FLX reads generated from two replicate SAGs mapped to the reference *Prochlorococcus* MED4 genome; of the few reads that did not match the reference, 94–99% either had best BLAST hits to human DNA or had no match in the NCBI nr database (Table S1). The presence of this small number of human sequences most likely resulted from contamination during either MDA or library construction, whereas unidentifiable reads could be template-independent byproducts of the amplification, such as primer dimers [8], [9]. In short, successive rounds of FACS – coupled with strict handling procedures – can virtually eliminate contaminating DNA from single-cell MDA reactions without sacrificing throughput.

### Single-cell genome recovery

The ultimate goal of single-cell genome amplification is to generate high-quality DNA template for complete genome sequencing and assembly. While previous attempts to sequence genomes from individual prokaryotic cells captured large portions of their genomes [4], [5], up to >30% of those genomes were thought to be missing. Furthermore, despite the advances reported in this study, it remains unclear whether complete genome recovery from individual cells is truly achievable. Incomplete genome retrieval may result from chromosomal breaks or DNA damage that lead to the loss of some genomic regions. In addition, stochastic processes in primer binding and amplification during the initial stages of MDA may cause uneven amplification across the genome, leaving some regions extremely abundant in sequencing libraries while others have little or no representation [2], [8], [9], [11].

**Figure 1:**
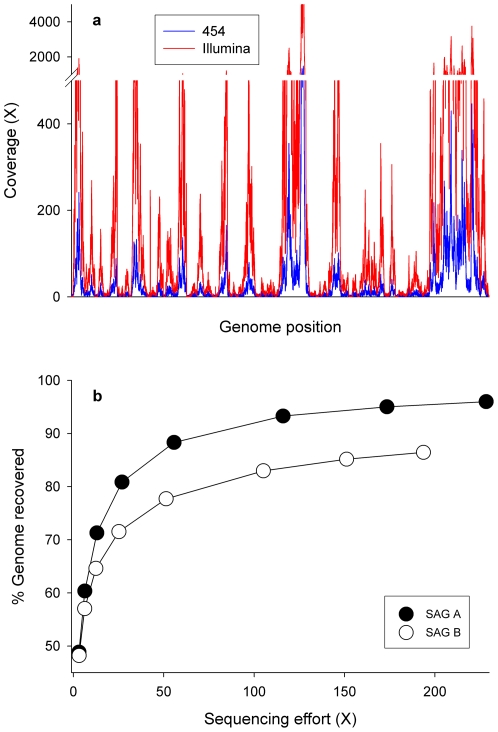
Sequencing coverage after WGA of individual *Prochlorococcus* cells. A) Average fold-coverage within 100 bp windows was determined for SAG A from the alignment of 454-FLX read data (blue) and Illumina read data (red) to the *Prochlorococcus* MED4 reference genome. B) Genome recovery for individual *Prochlorococcus* cells, SAG A and SAG B, as a function of sequencing effort for Illumina libraries (35 nucleotide read lengths). Each datapoint represents the mean genome recovery from at least two reference-guided assemblies. Standard deviations are too small to be visible on the figure.

To investigate what fraction of the *Prochlorococcus* MED4 genome could be recovered using our high-throughput pipeline, we selected two single-cell genomes for sequencing on both 454-FLX and Illumina platforms. As expected, the replicate *Prochlorococcus* SAGs A and B displayed large variations in local sequencing coverage (Figure 1a). Single-cell genome libraries were sequenced to an average depth of ∼40X with 454-FLX, but the representation of specific loci ranged from 0 to >2,500X, with a median coverage of 8.6X and 7.5X for SAGs A and B, respectively (Table S2). Despite this large variation in coverage, 97.6% and 74.7% of the SAG A and SAG B genomes were recovered at >1X coverage, respectively. Missing regions in the SAG A were spread across the genome, with most gaps being <40 bp, and the largest being 1,271 bp. By contrast, SAG B had larger gaps in coverage, with the greatest being 23,992 bp. When Illumina sequencing data were combined with the 454 reads, the fraction of genome recovered increased to 99.6% and 91.1% for SAGs A and B, respectively (Table S2).

These last results clearly demonstrate the benefits of sequencing to high coverage, although this strategy was inefficient at capturing previously unseen genomic regions. A plot of the genome recovery versus depth of sequencing revealed that recovery rapidly approached saturation in both libraries, with few additional sequencing reads mapping to missing regions (Figure 1b). Nonetheless, the virtually complete retrieval of a genome from a single *Prochlorococcus* cell confirms that generating draft-quality, if not completely closed, genomes is an achievable goal for single-cell genomics.

### Amplification bias

It has previously been suggested that MDA may show a bias against regions with high GC content [24]. Other studies [8], [10], [11], however, have presented evidence of random over-amplification, perhaps due to stochastic priming and amplification at the beginning of the MDA reaction. We decided to address the question of amplification bias more exhaustively by analyzing complete coverage maps for seven replicate *Prochlorococcus* MED4 SAGs.

**Figure 2:**
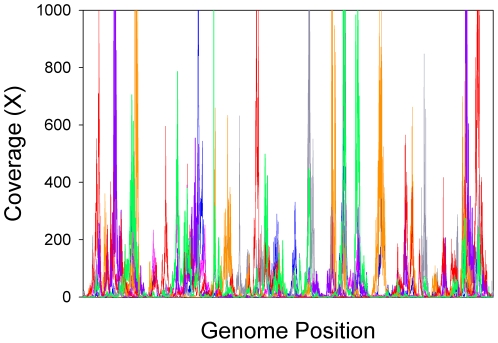
Variation in coverage depth among replicate single-cell WGA libraries. Coverage maps generated from Illumina sequencing data (35 nucleotides read lengths) for seven replicate *Prochlorococcus* single-cell amplified genomes (SAGs) are overlayed. SAG A (pink), SAG B (blue), SAG C (grey), SAG D (red), SAG E (green), SAG F (orange), and SAG G (purple).

Sequencing coverage varied >1000-fold over the whole genome in all replicate SAGs, but the over-represented regions differed among replicates. No clear patterns were discerned by overlaying coverage maps (Figure 2), or from pair-wise nonparametric correlations of coverage and genome position (Table S3). Kendall's W, a coefficient of nonparametric multiple correlation for all replicates, was estimated at 0.16 (p<0.01), indicating a small but statistically significant relationship may exist between genome position and high representation in the sequencing library. However, if some small bias towards particular regions does exist, it does not appear to be related to GC content. The GC content of *Prochlorococcus* MED4 ranged from 8% to 78%, with a mean of 31%, across the 100-bp windows analyzed in this study, yet nonlinear regression of GC content and coverage produced an R^2^<0.001. This observation notwithstanding, uneven amplification presents a significant challenge, and should be addressed in single-cell genomics development efforts.

### Library normalization

Uneven amplification during single-cell WGA results in a disproportionately large effort directed towards sequencing a relatively small fraction of the genome [11]. For example, 31% of all bases sequenced from the SAG A 454-FLX library aligned with only 2% of the genome. Reducing this large representational bias should increase sequencing efficiency and facilitate genome assembly. Using a custom designed microfluidic chip, Marcy *et al*. (2007) demonstrated that MDA reactions performed in nanoliter reactors produced a more even representation of loci amplified from a single-cell, but the impact on *de novo* assembly was not investigated. The addition of trehalose to MDA reactions may also produce more homogeneous MDA amplifications [25], but the method has not been specifically applied to single-cell amplification. We explored an alternative approach in which we reduced variations in gene abundance after amplification.

**Figure 3:**
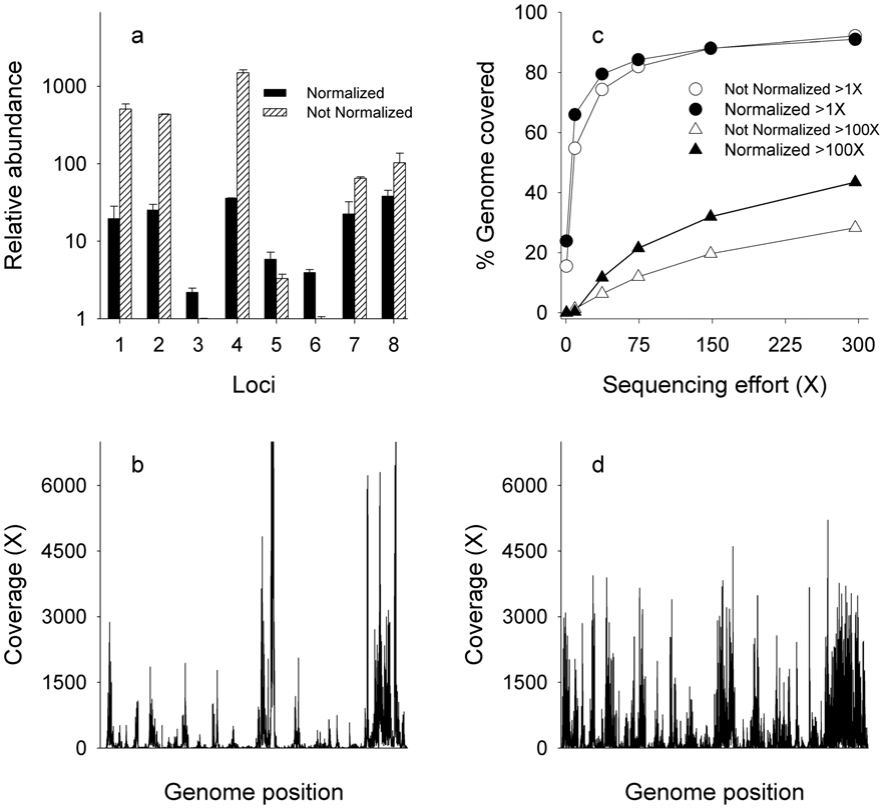
Impact of library normalization on coverage variation. A) The relative abundance of 8 loci distributed across the entire genome of SAG A before and after normalization. Error bars represent one standard deviation of three separate normalization reactions. B) Coverage depth of SAG A prior to normalization. C) Fraction of genome covered at >1X and >100X in normalized and non-normalized control libraries of SAG A. Error bars representing 95% confidence intervals are obscured by symbols. D) Coverage depth of SAG A after normalization.

To this end, we adapted a protocol developed for cDNA library normalization [26], in which highly abundant sequences are degraded by a duplex-specific nuclease based on their re-annealing kinetics. The technique can be applied to existing DNA libraries, does not require any specialized equipment, and is amenable to high throughput. The procedure was first tested by comparing the relative abundance of eight loci that were distributed across the complete genome and which fell in regions found to have both high and low sequence coverage in previously sequenced libraries of SAGs A and B (Figure 3a, Figure S2). Prior to normalization, the difference in abundance between the most- and least-abundant of the eight loci exceeded 1000-fold for SAG A and 2,500-fold for SAG B. After normalization, the discrepancy in relative abundance was reduced to approximately 20-fold in each library: highly abundant loci were drastically reduced while the ones with low initial representation typically saw an increase in their concentration after normalization (Figure 3a, Figure S2). Moreover, the overall variation among loci was comparable to the ratios obtained from MDA in nanoliter reactors [10].

Paired-end Illumina reads, each 71 nucleotides long, were generated to assess the impact of library normalization on coverage variation and genome recovery (Supplementary Note S1). As expected, the normalization procedure reduced coverage variation along the genome (p<0.01) compared to an non-normalized control, resulting in a more even distribution of the sequencing reads (Figure 3b,d). For example, at a sequencing effort of ∼300X, the average coverage of regions that were initially covered at >3,000X dropped from 10,643X to 1,690X. Conversely, the average coverage of regions that were initially covered at <30X increased from 8.8X to 32.5X. While a similar proportion of the genome was recovered in both the libraries at >1X, a larger proportion of the genome was covered at greater depth in the normalized library (Figure 3c). For example, 43% of the genome was covered at >100X in the normalized library, whereas only 28% was covered in the untreated control.

Post-amplification normalization decreases variability in genome coverage resulting from the MDA reaction, and represents a useful method for increasing sequencing efficiency of single-cell genomics projects. Similarly, DNA library normalization could also play an important role in the exploration of low-abundance diversity in other samples, such as metagenomic or metatranscriptomic libraries, by reducing the frequency of overrepresented sequences. In addition, this physical normalization procedure can be complemented – or to some degree substituted for – with bioinformatic methods for reducing highly abundant sequences (see below).

### *De novo* assemblies

Working with a previously sequenced isolate enabled a thorough assessment of *de novo* assembly of single-cell genomes. Using the *Prochlorococcus* MED4 reference genome as a guide, we compared *de novo* assemblies of 454 and Illumina sequence data to determine optimal sequencing and assembly strategies. We also analyzed, for the first time, the impact of chimeric sequences on *de novo* genome assembly.

**Figure 4:**
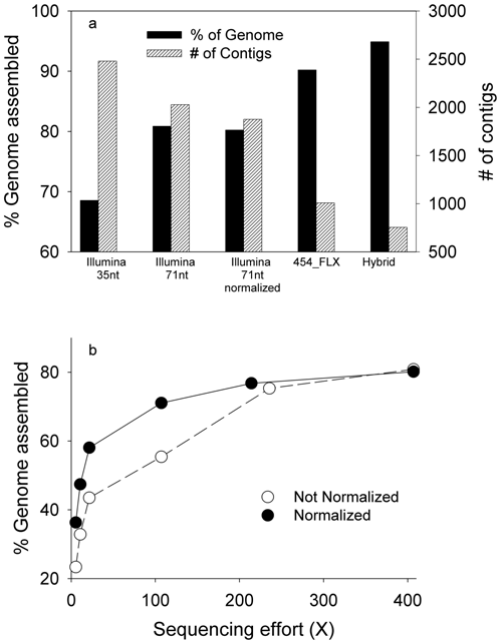
Comparison of *de novo* assembly results using 454-FLX and Illumina sequencing data. A) Fraction of genome (solid bars) and number of contigs (stripped bars) assembled *de novo* using various sequencing approaches. The sequencing efforts on each platform were: Illumina 35 nucleotides (nt) = 455X, Illumina 71nt = 407X, Illumina 71 nt normalized = 407X, 454-FLX = 39X, and Hybrid 454 and Illumina = 446X. B) Genome recovery from normalized and un-normalized Illumina 71 nt libraries as a function of sequencing effort.

The 454-FLX sequencing reads from SAG A generated a total of 1,008 contigs that accounted for 90% of the genome (Figure 4a, Table 1). The largest gap between contigs was 2,791 bp, the median gap size equaled 106 bp. The remaining, unrepresented 10% of the genome was distributed between small sections dispersed throughout the reference chromosome. By comparison, a smaller fraction of the genome was recovered from SAG B during *de novo* assembly, with 65% of the genome assembled into 830 contigs (Table 1). Eleven gaps in SAG B were >10 kbp in size. In comparison, *de novo* assemblies of unamplified DNA extracted from the initial *Prochlorococcus* MED4 culture performed significantly better, assembling 99.9% of the genome into 9 contigs. This discrepancy between genomic DNA extracted from a culture and the SAGs again demonstrates the impact of uneven amplification along the genome during WGA.

**Table 1:** *De novo* assembly statistics of single-cell amplified genomes.

Single-Cell Genome	Library Type	Sequencing Effort (X)	Number of contigs	% Genome Assembled	Largest Gap (bp)	Meidan Gap (bp)
SAG B	454-FLX	41	830	65	36,630	79
SAG A	454-FLX	39	1,008	90	2,792	106
SAG A	454-Illumina Hybrid	497	755	95	1,335	64

*De novo* assemblies using Illumina reads recovered a smaller fraction of the genome than their 454-FLX counterparts, reflecting the greater difficulty of assembling ultra-short reads. Using 35 nt-long reads, only 69% of the SAG A genome was obtained, in 2,480 contigs, while 57% of the SAG B genome was covered in 1,855 contigs. (Figure 4a, Table 1). In contrast, 99.9% of the unamplified *Prochlorococcus* culture was recovered in 660 contigs. Increasing the Illumina read lengths to 71 nucleotides enhanced genome recovery of SAG A to 81% while reducing the number of contigs to 2,028 (Figure 4a).

Post-amplification normalization substantially enhanced genome assembly when sequencing effort was <100X, but this effect was smaller at higher coverage (Figure 4b). For instance, a similar fraction of the genome was assembled from both normalized and non-normalized Illumina libraries with ∼400X coverage, although the normalized library generated 152 fewer contigs (Figure 4a). These assembly statistics alone do not entirely reflect the impact of normalization on genome assembly, however: *de novo* assembly of the non-normalized library was generally impossible without the prior removal of sequence reads from the most highly overrepresented regions, whereas assembly of the normalized library was possible without this additional step. A “bioinformatic normalization” procedure was thus necessary to assemble the library that did not undergo a physical normalization. We also found that regions with exceedingly high coverage were identified as “repeats”, and did not form contigs even when the assembly runs executed. Bioinformatic normalization facilitated assembly of these highly overrepresented regions. Importantly, our bioinformatic normalization approach required no prior information about the genome and could be used with organisms lacking a reference genome (see Methods for more details). In summary, post-amplification normalization improves sequencing efficiency and genome assembly, and in its absence, bioinformatic normalization may be required for *de novo* assembly.

We also investigated whether *de novo* genome assembly could be improved by combining the sequencing depth offered by the Illumina platform with the longer scaffolds provided by 454-FLX. This hybrid approach utilized the SAG A 454-FLX library, and reads from the physically normalized Illumina library. This strategy increased genome recovery to 95% (∼5% better than the 454-only assembly), and decreased the number of contigs from 1,008 to 769 (Figure 4a, Table 1). The addition of Illumina data also improved the accuracy of assembled contigs. For example, the frequency of mismatches and gaps in the alignment between contig sequences and the reference genome dropped significantly (p<0.01) from 0.24 mismatches/kbp and 0.70 gaps/kbp in the 454-only assembly, to 0.19 mismatches/kbp and 0.44 gaps/kbp in the hybrid assembly. The addition of Illumina data in the hybrid assembly most likely corrected erroneous SNPs and homopolymer length discrepancies in the 454 sequences – thereby reducing mismatches and alignment gaps – as has been demonstrated in previous studies combining 454 and Illumina data [27], [28],

### Chimeras and paired end sequencing reads

MDA has been shown to generate genome rearrangements, as evidenced by the occurrence of chimeric fragments [2], [11], presenting another potential obstacle to single-cell genomics by linking non-contiguous regions of a genome. However, the impact of chimeric sequences on *de novo* assembly had not been previously examined. In this study, we found 2–4% of reads in the 454-FLX single-cell libraries to be chimeric – equivalent to ∼1 chimera per 10 kbp (Table 2, Table S4). This rate of chimera formation, as well as the distance between joined regions, and the frequency of inversions (Table S4), are in good agreement with results from a previous study of single-cell MDA[2]. Higher rates of chimera formation have also been reported [11], but these resulted from additional chimera formation during a cloning step that is not used in generating 454 libraries.

**Table 2:** Chimera formation rates and impact on *de novo* genome assembly of 454-FLX reads.

Single-Cell Genome	Total Reads	Total Chimeras	Chimeras Assembled	Chimeras per 10 Kb	Correctly Assembled^a^	Incorrectly Assembled^b^	Chimeric Contigs
SAG A	294,514	5,907	3,039	0.92	2,797	242	7
SAG B	292,163	7,722	4,086	1.09	3,616	470	7

a chimeras split within 10 bp of identified chimeric junction

b chimeras not split within 10 bp of junction

In traditional studies, mate pairs from large-insert libraries play a crucial role in joining contigs into larger scaffolds. However, with an inherent MDA chimera formation rate of ∼1 per 10 kbp, the value of paired-end reads is reduced. For example, roughly 30% of clones would contain rearrangements in a standard 3 kbp-insert library; the frequency would be higher in larger insert libraries. Paired-end 454 “jumping” libraries appear to be even more problematic, since the library construction itself generates incorrectly joined inserts in 4–17% of paired reads (M.R. Henn & B.W. Birren, unpublished data). Indeed, a 2.7 kpb 454 paired-end library of SAG A contained 10,461 chimeric pairs and only 7,056 correct pairs. These data clearly indicate that paired-end sequencing reads must be used with caution when building scaffolds of single-cell MDA products.

Roche's assembly software [29], which allows reads to be split apart and assigned to different contigs, performed well with *de novo* assemblies of 454-FLX shotgun libraries. Roughly 94–96% of the chimeric sequences were either split correctly at the chimeric junction during assembly, or were not assembled at all. However, seven contigs in each of the single-cell libraries contained non-contiguous regions linked by chimeric reads (Table 2). The additional sequencing depth provided by the hybrid assembly of 454 and Illumina reads corrected 5 out of 7 of these misassembled contigs in SAG A, although two new chimeric contigs were formed. In one case only, several chimeric reads supported the incorrect assembly of two non-adjacent regions in one contig, possibly because a genome rearrangement occurred early during MDA. Under these circumstances, additional sequencing is unlikely to rectify the incorrect assembly, since chimeric sequences are far more abundant than correct sequences. Such a situation would be difficult to identify in assemblies of unknown organisms, but genome annotation may reveal problematic contigs in some cases. Overall, the impact of chimeric reads was relatively small when sequencing depth was high – as it was (∼450X) in the case of the combined 454-FLX and Illumina reads – but should not be considered negligible during *de novo* assembly of MDA products.

## Discussion

By combining FACS with semi-automated liquid handling, it is possible to rapidly generate hundreds of single-cell WGA libraries suitable for whole genome sequencing and assembly. Although these libraries show large variation in sequencing coverage, this variation can be mitigated by using a post-amplification normalization procedure, thereby increasing sequencing efficiency. We also demonstrate that with sufficient sequencing effort, virtually complete genomes can be recovered from individual bacteria.

Our results highlight the tremendous potential of single-cell WGA that exclusively uses second-generation sequencing technologies. They also highlight the challenges of *de novo* complete genome assembly, particularly from individual cells, even when virtually the entire genome is present in the sequencing libraries. Using longer sequencing reads should theoretically improve *de novo* assemblies, especially considering that most gaps between contigs in our study were relatively short. We computationally simulated 454 libraries with the same coverage as SAG A, but with read lengths of ∼500 bp, equivalent to current 454-Titanium libraries, which were not available at the time of this study. Over 98% of the genome was recovered in a *de novo* assembly of simulated ∼500 bp reads. A simulation with ∼700 bp reads, a recently achieved target (www.454.com), recovered 99% of the genome.

In addition to using longer reads, targeted primer walking approaches could also substantially improve *de novo* assemblies. Indeed, 99% of all missing regions in the hybrid assembly of SAG A were <600 bp, a distance that could be closed by a single Sanger sequencing read primed near the ends of each contig. Woyke *et al*. (2009) recently used several thousand primer walking reads to help assemble ∼90% of an uncultured *Flavobacterium* genome into just 19 contigs. In light of these results, it is reasonable to state that given sufficient effort, complete single-cell genome *de novo* assembly is possible.

Considering these advances, the challenges facing large-scale single-cell genomics are increasingly computational. For example, *de novo* assembly of SAGs does not capture all regions known to be present in a library based on reference-guided assembly. Minimizing the impact of chimeric reads remains another important challenge. While using assembly software that allows reads to be split can largely eliminate the influence of chimeric reads, non-contiguous regions can still be joined even when sequencing effort is high. New approaches, such as utilizing genome annotation to identify and correct chimeric contigs, will help address these issues in the future.

## Materials and Methods

### Cell sorting

The Influx cell sorter was prepared for ultra-clean sorting using procedures similar to those reported previously [12]. Briefly, fluidic lines were cleaned by running 10% bleach for 45 minutes, then rinsing with UV-treated ddH_2_0 for 20 minutes. Clean sheath fluid was generated by dissolving heat combusted NaCl (4 hours at 450°C) at a final concentration of 1% in ddH_2_0 that was UV-treated for 2 hours. The sheath fluid reservoir and sample collection tubes were also UV-treated for 2 hours before adding sheath fluid.

*Prochlorococcus* cells were sorted twice to dilute away extracellular DNA found in the culture. For example, ∼25,000 *Prochlorococcus* were sorted into 1.5 mL of clean sheath fluid during the first round of sorting. The same cells were sorted again, but this time sort droplets were deposited directly into reaction wells containing 3 µL of UV-treated PBS. Stringent sort conditions were used to ensure that only one cell was sorted into each reaction well. For example, the high rate of sort droplet formation (∼40,000/second) relative to the *Prochlorococcus* detection rate (∼3/second) ensured that no more than one cell was contained in each sort droplet. In addition, droplets containing *Prochlorococcus* were not sorted if any coincident particles were detected within 1.5 drop-lengths of the target cell.

### Single-cell genome amplification

Individual cells were deposited in wells of 96- or 384-well plates containing 3 µL of PBS buffer (137 mM NaCl, 2.7 mM KCl, 4.3 mM Na_2_HPO_4_,1.4 mM KH_2_PO_4_, pH 7.4), and lysed by adding 3 µL of solution A (0.13 N KOH, 3.3 mM EDTA pH 8.0, 27.7 mM DTT). The mixture was neutralized with 3 µL of solution B (0.13 N HCl, 0.42 M Tris-HCl pH 7.0, 0.18 M Tris-HCl pH 8.0). The amplification reaction was then initiated by adding 11 µL of a master mix (220 mM Tris-HCl pH 7.5, 275 mM KCl, 55 mM, MgCl_2_, 27.5 mM (NH_4_)_2_SO_4_, 26 mM DTT, 90.9 µM random hexamers [protected by phosphorothioate linkages on the last two nucleotides [2], 1 mM dNTPs, 0.2X SYBR Green 1 and 100 units of RepliPhi Phi29 DNA polymerase from Epicentre Biotechnologies). The reactions were incubated at 30°C for 10 hours during which SYBR green I fluorescence was measured every 6 minutes. The reaction was then heat-inactivated at 80°C for 10 minutes and immediately cooled to 4°C. The resulting DNA was diluted 100 fold in ddH_2_0 and PCR screened with primers (ITS-F: 5′-CCGAAGTCGTTACTYYAACCC-3′ and ITS-R 5′-TCATCGCCTCTGTGTGCC-3′), which target the internal transcribed spacer (ITS) of *Prochlorococcus*.

Selected cells were subjected to a second round of MDA amplification in order to obtain sufficient amounts of DNA to prepare sequencing libraires. Five microliters of the initial amplification were mixed with the appropriate buffer (40 mM Tris-HCl pH 7.5, 50 mM KCl, 10 mM MgCl_2_, 5 mM (NH_4_)_2_SO_4_ and 4 mM DTT), 50 µM phosphorothioate-protected random hexamers, 1 mM dNTPs, and 2000 units of RepliPhi Phi29 DNA polymerase. The reaction was incubated at 30°C for 10 hours and heat-inactivated as previously described. The resulting DNA was purified using Qiagen's QiaAmp DNA mini kit according to the manufacturer's supplementary protocol: “Purification of REPLI-g amplified DNA”.

### Preparation of sequencing libraries

454-FLX (454 Life Sciences) libraries were generated using three micrograms of genomic DNA. DNA was sheared using Adaptive Focused Acoustic technology (Covaris, Inc.) to generate DNA fragments of 400-1,500 bp in length. DNA fragments of approximately 400 bp were size selected using Solid Phase Reversible Immobilization (SPRI) bead capture (Agencourt Bioscience). Selected fragments were end-repaired and ligated to 454 sequencing adapters. Single stranded 454 libraries are then generated following methods previously described [29].

Paired-end “jumping” libraries for 454-FLX sequencing were generated from five micrograms of genomic DNA that was sheared to generate fragments of 2.5-3 kbp using the HydroShear technology (Genomic Solutions). Resulting fragments were end-repaired, column-purified and ligated with loxP adapters. Adapted fragments were then circularized using the loxP sites as a target for a Cre excision reaction using the Cre Recombinase enzyme. The circularized constructs containing the genomic DNA were column-purified and sheared using Adaptive Focused Acoustic technology (Covaris, Inc.). Sheared constructs were end-repaired and ligated with 454 paired end sequencing adapters. Single stranded 454 libraries were then generated, amplified on beads with emulsion PCR, and sequenced as previously described [29].

Libraries suitable for sequencing using the Illumina Genome Analyzer (Illumina, Inc.) were generated using a modified version of the standard Illumina GA protocol. Three micrograms of genomic DNA were used to generate libraries. Genomic DNA was sheared using Adaptive Focused Acoustic technology (Covaris, Inc.) to generate fragments of 100–300 bp in length. Fragments were size-selected by SPRI to ∼180 bp. Selected fragments were end-repaired, column-purified, tailed with an A nucleotide using Taq polymerase, and ligated with T nucleotide overhang Illumina forked paired end sequencing adapters (Illumina, Inc.). Libraries were then PCR amplified and sequenced as previously described [30].

### Sequencing library normalization

Roche 454-FLX libraries (400–1200 ng) were resuspended in 16 µL of hybridization buffer (50 mM Hepes pH 7.5, 500 mM NaCl) and heat denatured at 98°C for 2 minutes in a MJ Research PTC100 thermal cycler. The temperature was next slowly decreased to 65°C at a rate of 0.2°C/minute to allow complementary DNA strands to re-anneal. The temperature was maintained at 65°C for 90 minutes before 17 µL of pre-warmed 2X DSN buffer (100 mM Tris-HCl, pH 8.0, 10 mM MgCl2, 2 mM DTT) was added, and the reaction was incubated at 65°C for 10 minutes. One unit of Trimmer duplex-specific nuclease (Evrogen) was then introduced and the temperature was maintained at 65°C for another 25 minutes. The reaction was ended by adding 35 µL of stop solution (0.5 M EDTA pH 8.0) and incubating at 95° for 10 minutes in order to inactivate the enzyme. The single-stranded DNA mixture was then made double-stranded by performing PCR amplification with Roche-454-FLX primers (CCATCTCATCCCTGCGTGTCCCATCTGTTCCCTCCCTGTCTCAG and CCTATCCCCTGTGTGCCTTGCCTATCCCCTGTTGCGTGTCTC). The appropriate number of PCR cycles was determined by qPCR in order to stay in the log linear phase of the PCR amplification.

Normalization was initially assessed using qPCR to follow changes in the relative abundance of different loci within the reference genome. Eight loci were selected based on coverage depths observed in 454-FLX libraries of SAGs A and B. Specific primers were designed to quantify these loci, while primers matching 454-FLX adapter sequences were used to quantify the entire library. QPCR reactions consisted of 6 µL of 454 library as DNA template, 1.5 µL of primers (500 nM final conc.), and 7.5 µL of Quantitect SYBR Green master mixture (Qiagen). QPCR reactions were performed on Roche's Light Cycler 480 (melt for 30 seconds at 95°C, anneal for 30 seconds at 55°C, and extend for 30 seconds at 72°C; 50 cycles). Delta Ct values for each locus were determined relative to Ct values from reactions with primers matching the 454 adapters. Delta delta Ct values were calculated to determine the relative abundance of each locus before and after normalization.

Normalized 454-FLX libraries were converted to the Illumina format for sequencing (see Supplementary Note). This conversion to the Illumina paired-end format involved PCR amplification with deoxyuridine-containing oligonucleotide, treatment with the USER enzyme mixture (New England Biolabs), and treatment with S1 nuclease to remove 454-FLX adaptors from the library. Adaptors for paired-end Illumina sequencing were then ligated following standard conditions [30].

### Characterizing contaminants and chimeras

To examine carry-over of free DNA during sorting, a culture of *Prochlorococcus* MED4 was intentionally contaminated with a gel purified fragment of the pB1H1 plasmid (accession number DQ515894) at a final concentration of 5 ng/mL. This concentration is equivalent to free-DNA concentrations found in seawater. Cells from the contaminated culture were sorted twice to dilute away plasmid DNA, resulting in an estimated final concentration of ∼10^−3^ plasmids per reaction well. After performing MDA reactions, two replicate single-cell genomes were selected for sequencing on the Illumina platform. Illumina sequences (35 nucleotides) were mapped to the plasmid to identify potential contaminants using CLCBio Genomics workbench software package.

Contaminating sequences in the 454-FLX libraries were identified during reference assemblies to the *Prochlorococcus* MED4 genome. Reads marked as “unmapped” by Roche's gsMapper software were compared against the NCBI nr database using BLAST [31]. Sequences with best hits to organisms other than *Prochlorococcus*, as well as those with no hits to the database, were identified as contaminating sequences.

Chimeras were identified using a procedure described previously [2]. Reads marked as “partially mapped” by Roche's gsMapper software were compared to the *Prochlorococcus* MED4 genome using BLAST. Reads with two different regions that mapped to separate locations along the reference were identified as chimeras. Chimeric regions on of the 454 read also had be >20 bp long, not including an overlap of start/stop positions up to 10 bp. The impact of these chimeric reads on *de novo* genome assembly was determined by identifying contigs formed with chimeric reads. The gsAssembler software allows reads to by split apart and assembled into separate contigs, so output from 454Assembly.ace file was used to determine where chimeric reads were split during assembly. If reads were split >10 bp away from the chimeric junction, or not split at all, then the contigs containing these reads were compared against the reference genome. Contigs that did not align along their entire length were examined individually using Consed to determine if chimeric reads had joined non-contiguous genomic regions.

### Coverage variation and bias analysis

Coverage maps were generated with MAQ [32] for seven replicate single-cell genomes using Illumina sequence data (35 nucleotides), and average coverage values for 100 bp windows were calculated using a custom perl script. Average coverage values of each replicate were normalized to sequencing effort of SAG B, which had the fewest reads. Using MATLAB, pair-wise nonparametric correlation coefficients (Kendall tau), and a coefficient of nonparametric multiple correlation for all replicates (Kendall's W), were calculated for regions with >50X coverage in at least one replicate (n = 10,968 regions; p = 7 replicates). The Kendall's W statistic was transformed into a chi-square statistic (chi-square  =  p(n–1)W) to determine significance using the chi-square distribution. In addition, a nonlinear regression coefficient was calculated by fitting average coverage and GC content over 100 bp windows to a Gausian model using MATLAB. A Gaussian model would apply if amplification is biased against regions with either high or low GC content.

Average coverage values (100 bp windows) from normalized and un-normalized libraries of SAG A were also used to assess the impact of physical normalization on coverage variation. Differences in coverage variance between the libraries were examined with the Ansari-Bradley test using MATLAB. Coverage values were corrected by median subtraction prior to testing.

### Genome assembly

Roche's Newbler assembly software (gsMapper and gsAssembler) were used to perform reference-guided and *de novo* genome assembly, respectively, using 454-FLX sequence data. Alignment output from gsMapper provided gap positions in reference assemblies. Contigs from *de novo* assemblies were aligned to the reference genome using BLAST in order to determine gap positions.

For Illumina data, reference-guided assemblies were performed with MAQ [32]. *De novo* assemblies of short Illumina reads (35 nucleotides) were performed with Velvet [33] (k = 25), while longer Illumina reads (71 nucleotides) were performed with a combination of Velvet and gsAssembler. The gsAsssembler software was used with 71 nucleotides reads because it produced longer contigs and recovered a larger fraction of the genome than did Velvet. In these assemblies, batches of 500,000 Illumina reads were assembled *de novo* using gsAssembler, and contigs >2000 bp were split into shorter contigs (≤1,950 bp) with 100 bp overlaps. Illumina reads that did not form contigs with gsAssembler were pooled and assembled *de novo* into ≥100 bp contigs using Velvet. Shortened contigs generated by gsAssembler were pooled with contigs generated from Velvet for a final assembly with gsAssembler. The fraction genome recovered during from all *de novo* assemblies was confirmed using BLAST alignments to the *Prochlorococcus* MED4 reference genome.

Both 454-FLX and Illumina reads were used to produce a hybrid assembly of SAG A. Batches of 500,000 Illumina reads (71 nucleotides) from a normlized library of SAG A were assembled *de novo* using gsAssembler, and contigs >2000 bp were shortened as describe above. Illumina reads that did not form contigs with gsAssembler were pooled and assembled *de novo* into ≥100 bp contigs using Velvet. These Velvet contigs were combined with 454-FLX reads and shortened Illumina contigs for the final *de novo* assembly using gsAsssembler. Genome recovery was assessed using BLAST alignements to the reference genome.

The frequency of mismatches and gaps in the alignment between contig sequences and the reference genome was determined by BLAST. Data for comparing the frequency of alignment gaps and mismatches (i.e. gaps per kbp and mismatch per kbp) in different *de novo* assemblies were generated with bootstrap resampling (n = 1,000) using MATLAB. A Mann-Whitney U-test was performed to examine differences between 454-only and hybrid *de novo* assemblies.

### Bioinformatic normalization of Illumina data

Illumina reads (71 nucleotides) from over represented genomic regions were removed prior to genome assembly in order to reduce coverage variation along the genome. These regions were identified by first assembling 25,000 and 500,000 randomly selected reads into contigs using gsAssembler. To find the most over represented regions, the length of each contig was divided by the number of reads used to assemble the contig. In this case, contigs with a ratio of <1 bp/read were considered over represented. Next, all Illumina reads were mapped to these contigs using gsMapper. Reads that did not fully map to over represented regions were combined with the contigs covering these regions in order to produce a bioinformatically normalized dataset for further assembly. This normalization was necessary to prevent gsAssembler from stalling during assembly.

### Simulating long 454 reads

Using the reference genome as a guide, the length of each 454-FLX read in the SAG A library was extended to ∼500 bp and ∼700 bp from its 5′ end. This maintained both read orientation and relative coverage bias in the simulated data. Approximately one chimeric read was generated per 10 kbp. In addition, 80% percent of these chimeras joined regions <10 kbp apart on the genome, and 80% were inversions, as observed in the initial data (Table S4). Simulated reads were assembled using gsAssembler software.

## Supporting Information

Supplementary Note S1 454-FLX sequencing of normalized libraries
(0.02 MB DOC)

Table S1 Contamination levels in 454-FLX libraries. a =  no match to NCBI nr database
(0.01 MB PDF)

Table S2 Genome sequencing and coverage statistics for two single-cell genomes and an un-amplified culture of Prochlorococcus MED4. The 454-Illumina hybrid data include all 454-FLX, Illumina (35 nt), and Illumina (71 nt) reads.
(0.04 MB PDF)

Table S3 Pair-wise nonparametric correlation (Kendell tau) of genome position and coverage depth for seven replicate single-cell genomes (SAGs A-G). Genome positions with >50X coverage in at least one replicate were compared. * = p<0.05
(0.01 MB PDF)

Table S4 Chimera formation in 454-FLX libraries
(0.03 MB PDF)

Figure S1 Quantification of background contamination levels in whole genome amplification reactions. MDA amplification kinetics were monitored in real-time for different numbers of sorted cells -as well as empty sort droplets- according to the method of Zhang et al. (2006). The kinetics of the MDA amplification, monitored through SYBR green I fluorescence, is proportional to the amount of DNA present in the reaction well. Importantly, reactions containing empty sort droplet display a significant delay in their amplification, thus indicating lower DNA content (generally ranging 2 to 4 orders of magnitude lower than the DNA levels detected in reactions containing single-cells).
(0.04 MB PDF)

Figure S2 Impact of library normalization on SAG B. Relative abundance before and after normalization of 8 loci distributed across the entire genome and were found in high and low abundance regions of the SAG B library was measured by qPCR (see methods for details).
(0.24 MB PDF)
