# Supplementary material for: Whole Genome Amplification and De novo Assembly of Single Bacterial Cells
Source: PLoS One. 2009 Sep 2;4(9):e6864. doi: 10.1371/journal.pone.0006864 (PMC2731171; doi:10.1371/journal.pone.0006864)
Supplement: Supplementary Note S1 — 454-FLX sequencing of normalized libraries (0.02 MB DOC) [file pone.0006864.s001.doc]

**Supplementary Note**

**454-FLX sequencinng of normalized libraries**

Normalized libraries were initially sequenced on the 454-FLX platform but the average read length obtained was only 109 bp rather than the expected ~250 bp obtained with the un-treated library. The phenomenon was reproduced in at least two separate runs, and its exact cause is still under investigation. Some evidence suggest that many pyrosequencing reactions from normalized libraries might go out of phase, making base calling impossible after that point. In the meantime, the libraries were converted to the Illumina format and, although the reads were relatively short (71 bp), no difference could be seen between the normalized library and the un-treated control.
