# Supplementary material for: Whole Genome Amplification and De novo Assembly of Single Bacterial Cells
Source: PLoS One. 2009 Sep 2;4(9):e6864. doi: 10.1371/journal.pone.0006864 (PMC2731171; doi:10.1371/journal.pone.0006864)
Supplement: Table S1 — Contamination levels in 454-FLX libraries. a = no match to NCBI nr database (0.01 MB PDF) [file pone.0006864.s002.pdf]

Supplementary Table 1: Contamination levels in 454-FLX libraries.

Single-Cell Genome	Total Reads	Non-Target Reads	Human	Other	Unidentified[a]
SAG A	294,514	2,899	687	26	2,186
SAG B	292,163	546	364	30	152

a= no match to NCBI nr database
