# Supplementary material for: Whole Genome Amplification and De novo Assembly of Single Bacterial Cells
Source: PLoS One. 2009 Sep 2;4(9):e6864. doi: 10.1371/journal.pone.0006864 (PMC2731171; doi:10.1371/journal.pone.0006864)
Supplement: Table S2 — Genome sequencing and coverage statistics for two single-cell genomes and an un-amplified culture of Prochlorococcus MED4. The 454-Illumina hybrid data include all 454-FLX, Illumina (35 nt), and Illumina (71 nt) reads. (0.04 MB PDF) [file pone.0006864.s003.pdf]

Supplementary Table 2. Genome sequencing and coverage statistics for two single-cell genomes and an un-amplified culture of *Prochlorococcus* MED4.
The 454-Illumina hybrid data include all 454-FLX, Illumina (35nt), and Illumina (71nt) reads.

	Un-amplified Culture	SAG A	SAG B
**Roche 454-FLX**			
Sequencing effort (X)	40.0	38.5	42.8
Median coverage (X)	38.2	8.60	7.50
% genome recovered ≥ 1X	99.9	97.6	74.7
# of gaps	5	546	569
Largest gap (bp)	720	1,271	23,992
Median gap length (bp)	180	40	196
**Illumina (35nt)**			
Sequencing effort (X)	52.0	228	194
Median coverage (X)	52.0	39.5	37.4
% genome recovered ≥ 1X	99.9	96.0	86.5
# of gaps	2	1472	2,443
Largest gap (bp)	4	1433	10,209
Median gap length (bp)	3	19	24
**454-Illumina Hybrid**			
Sequencing effort (X)	92.0	1,353	667
Median coverage (X)	92.3	274	125
% genome recovered ≥ 1X	99.9	99.6	91.1
# of gaps	1	210	1,152
Largest gap (bp)	3	324	5,290
Median gap length (bp)	3	18	31
