# Supplementary material for: Whole Genome Amplification and De novo Assembly of Single Bacterial Cells
Source: PLoS One. 2009 Sep 2;4(9):e6864. doi: 10.1371/journal.pone.0006864 (PMC2731171; doi:10.1371/journal.pone.0006864)
Supplement: Table S3 — Pair-wise nonparametric correlation (Kendell tau) of genome position and coverage depth for seven replicate single-cell genomes (SAGs A-G). Genome positions with >50X coverage in at least one replicate were compared. * = p<0.05 (0.01 MB PDF) [file pone.0006864.s004.pdf]

Supplementary Table 3: Pair-wise nonparametric correlation (Kendell tau) of genome position and coverage depth for seven replicate single-cell genomes (SAGs A-G). Genome positions with >50Xcoverage in at least one replicate were compared. * = $p<0.05$

	G	F	E	D	C	B	A
A	0.12*	-0.19*	0.22*	0.02*	-0.16*	0.07*	1
B	0.00*	0.11*	0.18*	-0.04*	0.01	1	
C	-0.08*	0.02*	0.07*	-0.01*	1		
D	0.17*	-0.09*	-0.01	1			
E	0.11*	0.03*	1				
F	-0.01*	1					
G	1						
