# Supplementary material for: Whole Genome Amplification and De novo Assembly of Single Bacterial Cells
Source: PLoS One. 2009 Sep 2;4(9):e6864. doi: 10.1371/journal.pone.0006864 (PMC2731171; doi:10.1371/journal.pone.0006864)
Supplement: Table S4 — Chimera formation in 454-FLX libraries (0.03 MB PDF) [file pone.0006864.s005.pdf]

Supplementary Table 4: Chimera formation in 454-FLX libraries

Single-Cell Genome	Total Reads	Total Chimeras	Chimeras joining regions <10kb apart	Inversions	Chimeras per 10 Kb
SAG A	294,514	5,907	4,784	4,886	0.92
SAG B	292,163	7,722	6,563	6,475	1.09
