# Supplementary figures and images for: Whole Genome Amplification and De novo Assembly of Single Bacterial Cells
Source: PLoS One. 2009 Sep 2;4(9):e6864. doi: 10.1371/journal.pone.0006864 (PMC2731171; doi:10.1371/journal.pone.0006864)

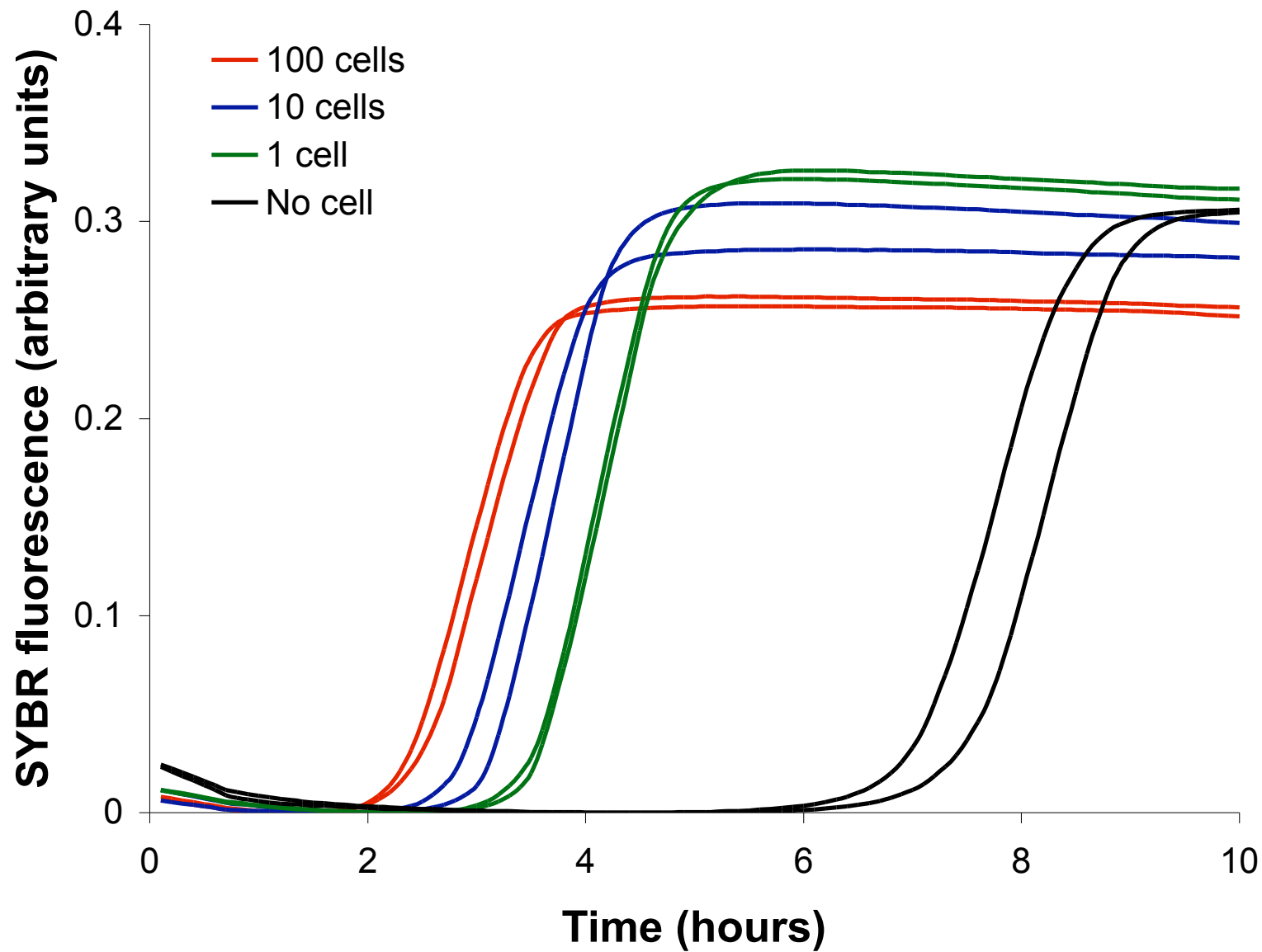

Supplement: Figure S1 — Quantification of background contamination levels in whole genome amplification reactions. MDA amplification kinetics were monitored in real-time for different numbers of sorted cells -as well as empty sort droplets- according to the method of Zhang et al. (2006). The kinetics of the MDA amplification, monitored through SYBR green I fluorescence, is proportional to the amount of DNA present in the reaction well. Importantly, reactions containing empty sort droplet display a significant delay in their amplification, thus indicating lower DNA content (generally ranging 2 to 4 orders of magnitude lower than the DNA levels detected in reactions containing single-cells). (0.04 MB PDF) [file pone.0006864.s006.pdf]

Suplemenatry Figure 2

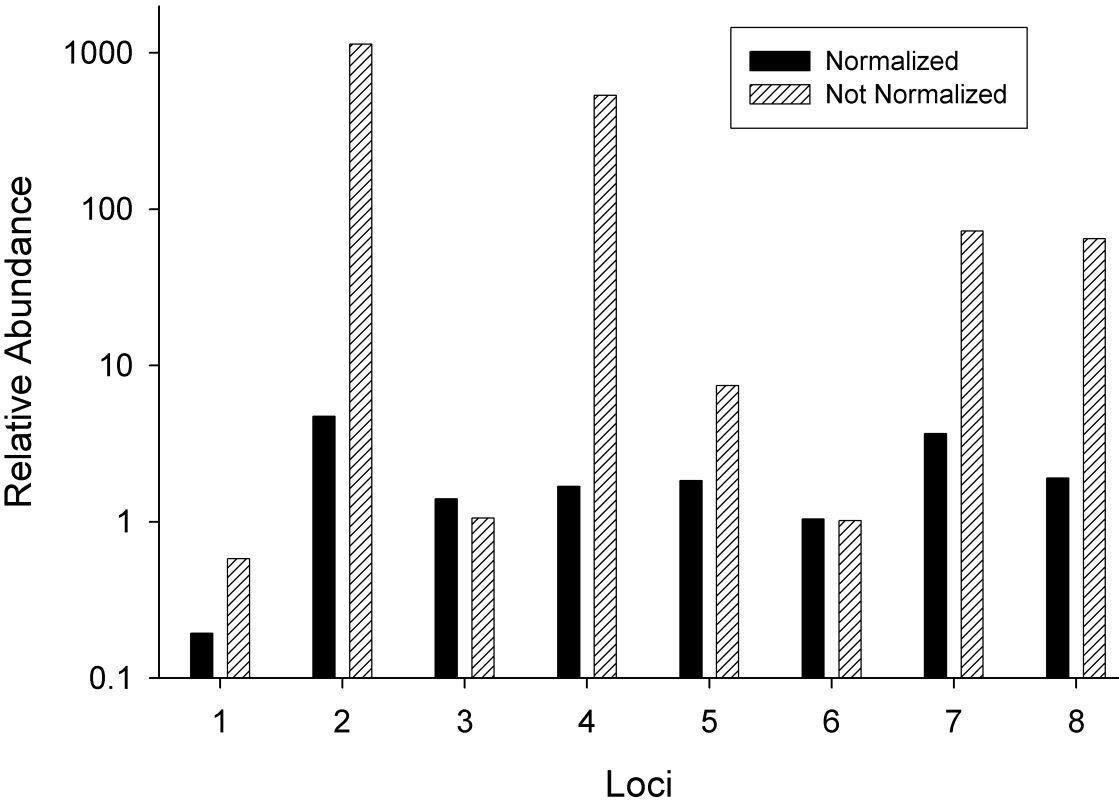

Supplement: Figure S2 — Impact of library normalization on SAG B. Relative abundance before and after normalization of 8 loci distributed across the entire genome and were found in high and low abundance regions of the SAG B library was measured by qPCR (see methods for details). (0.24 MB PDF) [file pone.0006864.s007.pdf]
